# Anomalous origin of the right coronary artery originating from the pulmonary trunk: pre- and postoperative computed tomography images and virtual reality reconstructions

**DOI:** 10.1097/MCA.0000000000001315

**Published:** 2023-12-29

**Authors:** Alexander Suchodolski, Michał Gałeczka, Mariola Szulik, Roland Fiszer, Jan Głowacki

**Affiliations:** a Doctoral School of the Medical University of Silesia; b Department of Cardiology, Congenital Heart Diseases and Electrotherapy, Silesian Center for Heart Diseases, Faculty of Medical Sciences in Zabrze, Medical University of Silesia; c Department of Congenital Heart Defects and Pediatric Cardiology, Silesian Center for Heart Diseases, Faculty of Medical Sciences in Zabrze, Medical University of Silesia, Katowice; d Department of Medical and Health Sciences, Faculty of Applied Sciences, WSB University, Dąbrowa Górnicza; e Department of Radiology and Radiodiagnostics, Faculty of Medical Sciences in Zabrze, Medical University of Silesia, Katowice; f Computed Tomography Laboratory, Silesian Centre for Heart Diseases, Zabrze, Poland

An asymptomatic 6-year-old boy was admitted after a murmur was detected during a routine medical examination. Transthoracic echocardiography showed left coronary artery (LCA) dilatation to 6.5 mm (z-score +7.54, normal: 1.64–3.65), no right coronary artery (RCA) originating from the right coronary cusp, collateral vessels seen over the cardiac apex, and inappropriate diastolic inflow to the main pulmonary artery. Cardiac computed tomography angiography was performed, which showed that the LCA was the only vessel arising from the aortic root and was proximally widened up to 6 mm. The trunk of the LCA was divided into a well-developed circumflex artery with a typical course, giving off the posterior descending artery branch and a very dilated left anterior descending artery (LAD). The RCA did not depart from the aortic root; instead, it arose from the pulmonary trunk. The width of the opening of the vessel was up to 6.4 mm (Fig. 1a,c,d and Supplementary Video 1, Supplemental digital content 1, http://links.lww.com/MCA/A624). The patient was diagnosed with an anomalous origin of the RCA from the pulmonary artery. Surgical correction was performed. Cardiac computed tomography angiography was obtained after 2 months and revealed a reduction of the LCA diameter (to 4.3 mm) and LAD diameter. RCA remained widened (5.6 mm). Collateral circulation diminished. (Fig. 1b,e and Supplementary Video 2, Supplemental digital content 2, http://links.lww.com/MCA/A625) The patient remained asymptomatic and demonstrated good clinical status during a 5.5-year follow-up.

**Fig. 1:**
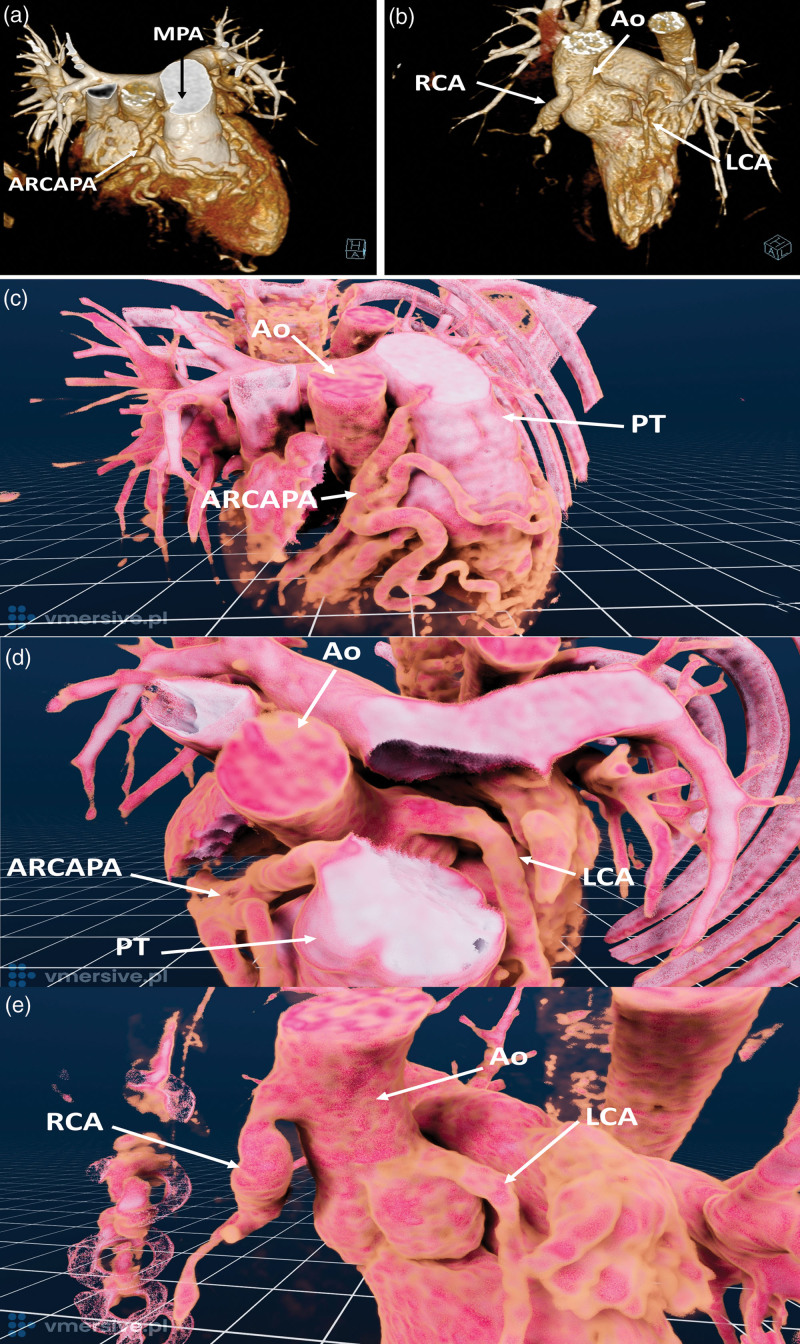
Volume Rendering Technique (VRT) of ARCAPA (a) before and (b) after correction. Two-dimensional picture of a three-dimensional virtual reality reconstruction of ARCAPA (VMersive software): (c) before the correction (d) RCA and LCA origin with the pulmonary trunk digitally removed for better visualization (e) after the corrective procedure. ARCAPA, anomalous origin of the RCA from the pulmonary artery; LCA, left coronary artery; RCA, right coronary artery.

## Acknowledgement

### Conflicts of interest

There are no conflicts of interest.

## Supplementary Material

